# Trends in Research Payments From Industry to Dermatologists

**DOI:** 10.7759/cureus.94824

**Published:** 2025-10-17

**Authors:** Craig Cronin, Aleksandra Drmanovic, Zeyad Hammadeh, Aurora J Grutman, Joseph G Cheaib, Zhuo T Su, Bruce J Trock, Gerard F Anderson, Misop Han

**Affiliations:** 1 James Buchanan Brady Urological Institute, Johns Hopkins University School of Medicine, Baltimore, USA; 2 Department of Health Policy and Management, Johns Hopkins Bloomberg School of Public Health, Baltimore, USA

**Keywords:** centers for medicare and medicaid services (cms), dermatology, industry-sponsored research payments, industry-sponsored research payments to dermatologists, non-covered entities, open payments program (opp), physician payments sunshine act (ppsa)

## Abstract

Introduction: The Open Payments Program (OPP) requires manufacturers to report payments to various entities involving physicians, aiming to increase transparency in industry-physician financial relationships. While general payments have been extensively studied, research payments, particularly those to non-covered entities (NCEs), remain poorly studied. Dermatology, a rapidly evolving specialty with significant industry-sponsored research activity, offers an ideal case to examine trends in research payments.

Methods: This was a record-based longitudinal study using research payment data from dermatologists in 2015-2023 in the OPP database. Both direct research payments (payments directly to physicians) and associated research payments (payments to teaching hospitals and NCEs with physician principal investigators (PIs)) were included. Payments were inflation-adjusted to 2023 U.S. dollars using the Consumer Price Index for All Urban Consumers (CPI-U).

Results: Between 2015 and 2023, 1,610 dermatologists received a total of $1.44 billion in research payments, with an average of 84% directed to NCEs with a dermatologist PI. Research payments to NCEs with a dermatologist PI declined from $161.0 million in 2015 to $107.6 million in 2023, but the overall trend was not statistically significant (p = 0.27). The top 10 NCE organizations received $179 million, with most being private institutions involved in pharmaceutical clinical trials. Four pharmaceutical companies accounted for nearly half of all research payments.

Conclusion: Most research payments from the industry to dermatologists originated from a small number of pharmaceutical companies and were directed to NCEs, which have minimal reporting requirements. These findings underscore gaps in transparency within the OPP and highlight the need for new disclosure requirements regarding the distribution and use of funds to NCEs.

## Introduction

To improve transparency in the relationships between the industry and healthcare providers, the Physicians Payments Sunshine Act (PPSA), part of the 2010 Affordable Care Act, mandated that manufacturers of medical products and group purchasing organizations (GPOs) report financial transactions with physicians, advanced practice clinicians, or teaching hospitals through the Open Payments Program (OPP) [1]. Established in 2013 by the Centers for Medicare and Medicaid Services (CMS), the OPP categorizes payments as general payments (e.g., gifts, meals, and speaking fees), ownership interests, or research payments [1].

Prior studies using the OPP database have demonstrated that industry payments influence prescribing behaviors and clinical research outcomes [2-5]. Research payments from the industry are understudied compared to general payments, even though they comprise over 50% of total payments reported to the OPP [6]. These research payment transactions are directed to either 1) covered recipients, such as teaching hospitals, physicians, and advanced practice clinicians, or 2) non-covered entities (NCEs), which include non-teaching hospitals and universities, as well as private research centers and contract research organizations (CROs), all with physician principal investigators (PIs). 

Dermatology represents an ideal case study for examining industry-sponsored research payments due to its rapid therapeutic innovation, high-volume outpatient care, and extensive industry collaboration in clinical trials, particularly for new biologic agents and small molecule therapies [7]. The specialty has received substantial pharmaceutical investment, growing private equity (PE) involvement, and evolving practice structures that create diverse pathways for industry-physician research partnerships. While prior studies have assessed general payments to dermatologists, research payments remain understudied, with no longitudinal analyses to date [4,8-13]. This study addresses this gap by evaluating the distribution and temporal trends of research payments to dermatologists from 2015 to 2023. By characterizing research payments by company, recipient, and associated product, we aim to provide clearer insights into financial relationships between the industry and dermatologists in the U.S.

## Materials and methods

Data source 

Research payments from 2015 to 2023 using the OPP database were adjusted to 2023 U.S. dollars using the Consumer Price Index for all Urban Consumers (CPI-U). We included both direct payments to physicians and associated research payments (i.e., payments to teaching hospitals and NCEs with physician PIs). In this study, exposures were defined as recipient type, dermatologist gender, and year of payment receipt, while outcomes included industry research payment metrics such as total payment value, number of payments, and per-recipient payment information from 2015 to 2023.

Payments to NCEs are only reportable if a covered physician or advanced practice clinician acts as the PI [14]. For reportable payments with multiple PIs (≤5), we attributed the full amount to the primary PI to avoid duplication. These payments were then stratified by PI gender and affiliation. Physicians were identified using National Provider Identifier (NPI) records. Individuals with single or multiple specialties were included if the primary taxonomy code matched dermatology codes defined by the National Uniform Claim Committee (NUCC). The taxonomy codes included were “Dermatology Physician” (207N00000X), “Clinical and Laboratory Dermatological Immunology Physician” (207NI0002X), “Dermatopathology Physician” (207ND0900X), “MOHS-Micrographic Surgery Physician” (207ND0101X), “Pediatric Dermatology Physician” (207NP0225X), and “Procedural Dermatology Physician” (207NS0135X). This study followed the Strengthening of the Reporting of Observational Studies in Epidemiology (STROBE) guidelines [15].

Industry payment categories 

Research payments from the industry were aggregated to the parent company, including contributions from both subsidiary and parent companies. A covered product is any drug, device, biological, or medical supply that is reimbursable by Medicare, Medicaid, or the Children’s Health Insurance Program that, for drugs and biologicals, requires a prescription [1]. Research payments associated with non-covered products were included in total research payment calculations, but excluded from product-specific analyses.

Statistical analyses 

Percentage changes were calculated using data from 2015 to 2023. All statistical tests were two-sided, with statistical significance set at a p-value of <0.05. Trends in the number of payments and their value were tested with linear regression models. Payment values per dermatologist over time were evaluated using a generalized linear regression model. A gamma distribution and log link were used to address the positively skewed payment data, which is common in financial datasets where few individuals receive much higher payments [14]. Clustering by physician accounted for repeated measures over time. Analyses were conducted using Stata version 18.0 (Stata Corp LLC, College Station, TX).

## Results

From 2015 to 2023, 1,610 dermatologists received $1.4 billion in research payments, with an average of 84% directed to NCEs with a dermatologist PI. Total annual research payments declined from $190.5 million in 2015 to $130.5 million in 2023, but this trend over time was not statistically significant (p = 0.23). Research payments to covered teaching hospitals accounted for 11% of all research payments, with a 4% increase from 2015 to 2023 that was not statistically significant (p = 0.07). Annual research payments to NCEs with a dermatologist PI decreased from $161.0 million in 2015 to $107.6 million in 2023, but the overall trend was not statistically significant (p = 0.27) (Table 1).

**Table 1:** Annual and overall trends in research payments to covered recipients including teaching hospitals, physicians, and non-covered entities (NCEs) with a dermatologist as the primary principal investigator (PI), 2015-2023 * p-values were obtained via a linear regression. All payment values were inflation-adjusted to 2023 United States dollars using the Consumer Price Index for All Urban Consumers: Medical Care in U.S. City Average (https://fred.stlouisfed.org). Abbreviations: β1, estimated slope from linear regression; CI, confidence interval; PI, principal investigator Number of unique dermatologists receiving research payments including direct/indirect 2015-2023 = 1,610

	Research payment value in million United States dollars (% of total research payment values)													
Recipient type	2015	2016	2017	2018	2019	2020	2021	2022	2023	Total	% change	β1	95% CI	p-value*
Covered teaching hospitals	18.8	15.9	14.2	16.3	17.8	19.1	17.9	21.3	19.6	161.1	4%	0.49	-0.05, 1.03	0.07
	(10%)	(9%)	(11%)	(11%)	(8%)	(11%)	(13%)	(15%)	(15%)	(11%)				
Covered physicians	10.7	11.0	9.8	11.1	12.0	6.1	3.6	2.31	3.38	69.94	-68%	-1.21	-1.8, -0.5	<0.01
	(6%)	(6%)	(8%)	(7%)	(6%)	(4%)	(3%)	(2%)	(3%)	(5%)				
NCEs with a dermatologist as the PI	161.0	147.3	104.4	122.2	185.0	147.6	119.7	118.7	107.6	1,213.7	-33%	-4.00	-12, 3.9	0.27
	(85%)	(85%)	(81%)	(82%)	(86%)	(85%)	(85%)	(83%)	(82%)	(84%)				
Total	190.5	174.2	128.4	149.7	214.9	172.9	141.2	142.4	130.5	1,444.7	-31%	-4.77	-3.6, 13.4	0.23

Between 2015 and 2023, 1,286 of the 1,610 dermatologists receiving research payments were PIs employed by NCEs. The median value of research payments to NCEs with a dermatologist PI remained stable over time (p = 0.31), increasing from $51,295 in 2015 to $55,311 in 2023. The number of male dermatologist PIs decreased significantly by 12% (p < 0.01), while the number of female PIs increased significantly by 28% (p < 0.05). Total payments to male dermatologist PIs declined from $115.9 million in 2015 to $66.4 million in 2023, although this change was not statistically significant (p = 0.07). Median payment values did not change significantly over time for either gender, increasing by 5% for males (p = 0.53) and 6% for females (p = 0.23) from 2015 to 2023. Across all years, female PIs consistently received lower median payments than male PIs (Table 2).

**Table 2:** Annual and overall trends in the receipt of and value of research payments with a dermatologist as the primary principal investigator (PI) from 2015 to 2023, stratified by gender of the primary PI * p-values for yearly trends in changes in the total and maximum payment values were obtained via linear regression, and p-values for yearly trends in payment values per physician PI were obtained via linear regression with a generalized estimating equation framework accounting for clustered physician effects and a gamma link to account for positively skewed payment data. All payment values were inflation-adjusted to 2023 USD values using the Consumer Price Index for All Urban Consumers: Medical Care in the U.S. City Average (https://fred.stlouisfed.org). Abbreviations: β1, estimated slope from linear regression; CI, confidence interval; IQR, interquartile range; USD, United States dollar Number of unique dermatologists receiving research payments under non-covered entities 2015-2023 = 1,286

Male dermatologist PIs	2015	2016	2017	2018	2019	2020	2021	2022	2023	Total	% change	β1	95% CI	p-value*
# of dermatologists as primary PIs for NCEs receiving payments	407	416	394	389	403	400	370	342	357	762	-12%	-7.65	-11.9, -3.3	<0.01
Payment value, million USD	115.9	106.2	76.2	84.2	125.1	99.9	76.1	68.5	66.4	818.5	-43%	-4.92	-10.3, 0.5	0.07
Median payment value per PI (IQR), USD	53,830 (9,007 - 231,231)	42,720 (7,915 - 222,175)	42,715 (8,987 - 169,983)	31,848 (5,888 - 222,963)	67,743 (9,597 - 294,978)	64,162 (11,946 - 235,165)	45,306 (12,183 - 216,800)	45,610 (10,929 - 176,607)	56,553 (11,960 - 189,750)	-	5%	951	-2,689, 4,591	0.53
Female dermatologists as PIs	2015	2016	2017	2018	2019	2020	2021	2022	2023	Total	% change	β1	95% CI	p-value*
# of dermatologists as primary PIs for NCEs receiving payments	197	208	217	234	283	270	261	247	253	524	28%	7.75	1.2, 14.3	<0.05
Payment value, million USD	45.2	41.1	28.2	38.1	60.0	47.8	43.6	50.2	41.2	395.4	-9%	0.86	-1.8, 3.6	0.48
Median payment value per PI (IQR), USD	43,795 (6,643 - 228,196)	26,501 (8,702 - 162,032)	33,043 (6,678 - 104,444)	31,783 (8,583 - 138,339)	45,981 (7,172 - 179,480)	58,928 (10,762 - 202,367)	39,780 (8,357 - 127,380)	39,737 (8,620 - 196,883)	46,354 (13,250 - 159,651)	-	6%	1,509	-1,332, 4,350	0.23

**Figure 1:**
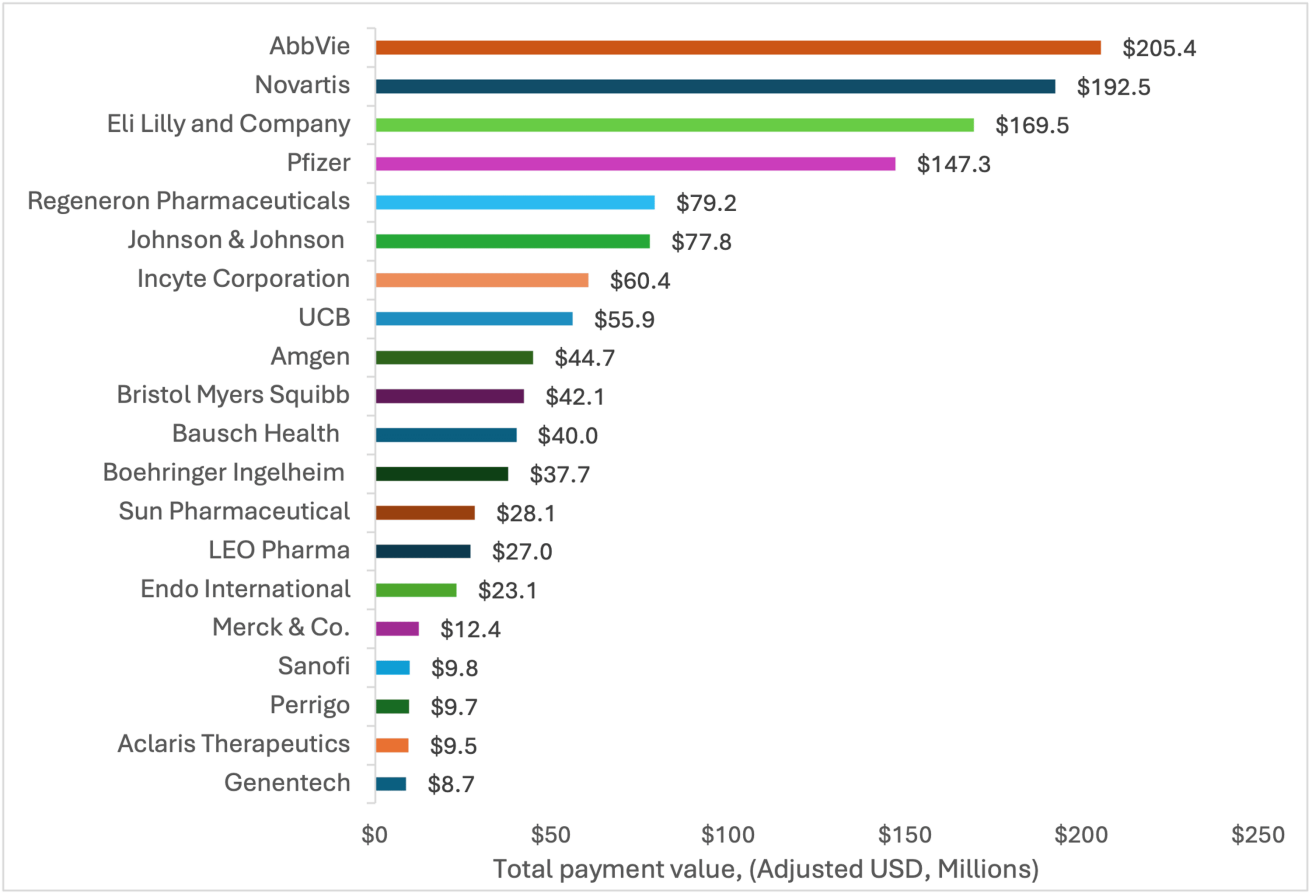
Value of research-specific payments for top 20 manufacturers (adjusted USD, millions) between 2015 and 2023

**Figure 2:**
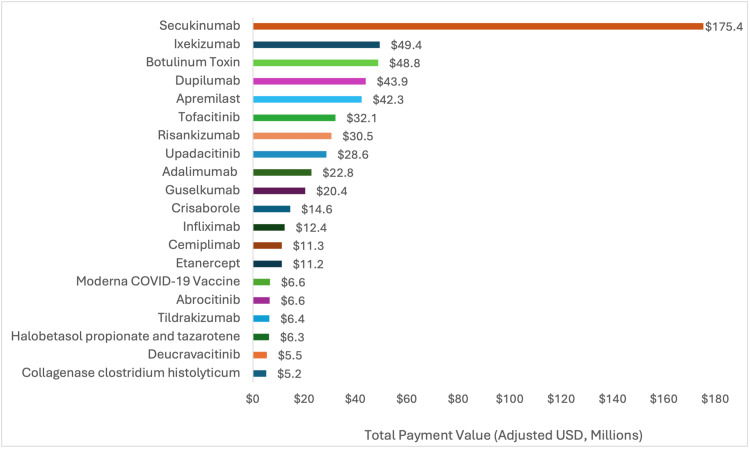
Value of research-specific payments for top 20 products (adjusted USD, millions) between 2015 and 2023

Four companies accounted for 49% of all research payments in dermatology, with the largest payments from AbbVie ($205.4 million), Novartis ($192.5 million), Eli Lilly ($169.5 million), and Pfizer ($147.3 million) (Figure 1). Half of all research payments (50.4%) did not have a reported product. Among payments with reported products, monoclonal antibodies for psoriasis (secukinumab and ixekizumab) were associated with the highest payments at $224.75 million. Secukinumab alone accounted for $175.35 million (Figure 2). Nine of the top 20 products were monoclonal antibodies, and four were small-molecule inhibitors.

From 2015 to 2023, the Hospital of the University of Pennsylvania ($18.8 million), the University of Michigan Hospital ($14.8 million), and the University of Texas MD Anderson Cancer Center ($9.0 million) received the highest teaching hospital payments (Table 3A). Among NCEs, the top 10 organizations received $179.1 million, with the Icahn School of Medicine at Mount Sinai ($43.7 million), the Center for Clinical Studies ($28.0 million), and the Oregon Medical Research Center ($24.8 million) receiving the most payments (Table 3B).

**Table 3:** Value of associated research payments to the top 10 teaching hospitals and non-covered entities (NCEs), representing the sum of payments from 2015 to 2023 (USD, millions) All payment values were inflation-adjusted to 2023 USD values using the Consumer Price Index for All Urban Consumers: Medical Care in U.S. City Average (https://fred.stlouisfed.org). Abbreviations: USD, United States dollar

A. Teaching hospitals with the highest value of associated research payments		B. Non-covered entities (NCEs) with the highest value of associated research payments	
Teaching hospital	Associated research payment value, million USD	NCEs	Associated research payment value, million USD
Hospital of the University of Pennsylvania	18.8	Icahn School of Medicine at Mount Sinai	43.7
University of Michigan Hospitals & Health Centers	14.8	Center for Clinical Studies	28.0
University of Texas MD Anderson Cancer Center	9.0	Oregon Medical Research Center	24.8
University of Alabama Hospital	8.1	FWD Clinical Research	23.8
Oregon Health & Science University Hospital and Clinics	7.8	Dermatology Research Associates	21.8
University of California, San Francisco - Langley Porter Psychiatric Hospital	7.7	TKL Research	9.3
University of California, Irvine Medical Center	6.2	Virginia Clinical Research	7.6
Rush University Medical Center	5.7	Progressive Clinical Research	7.0
Northshore University Health System	5.0	Tufts Medical Center	6.8
Tufts Medical Center	4.9	Premier Clinical Research	6.3

## Discussion

This study provides the first longitudinal analysis of research payments from the industry to dermatologists and the first to detail research payments to NCEs. Research payments to NCEs with a dermatologist PI declined from $161.0 million in 2015 to $107.6 million in 2023, representing an average of 84% of total research funding over the study period. Several of the top 10 NCE organizations receiving research payments were private institutions involved in clinical trials. Payments were also concentrated among a few leading pharmaceutical companies and primarily targeted biologic monoclonal antibodies and small-molecule inhibitors for atopic dermatitis and psoriasis; however, half of all research payments were not attributed to a covered product. These findings have broader implications for ongoing efforts to enhance transparency of the OPP database and safeguard research, education, and clinical judgment from undue influence.

While research payments to NCEs with a dermatologist PI declined from 2015 to 2023, they still accounted for 82% of all research payments in 2023. This contrasts with another study that noted a 23% increase in similar research payments from the industry to other medical specialties from 2015 to 2022 [14]. The overall decline in payments to dermatologist PIs in our study becomes clearer when stratified by gender. While females comprise 52% of the field, which is an increase from 41% over the last 15 years, our data demonstrated that females received just 33% of research payments to NCEs with a PI over the study period [16]. Similar gender disparities have been seen across specialties, suggesting a broader trend [17-19].

Market saturation in blockbuster drug indications, illustrated here by atopic dermatitis and psoriasis, may also explain the decline in research payments to NCEs with a dermatologist PI. Companies may be choosing to shift funding from clinical research toward general payments to support product promotion as markets become more concentrated [20-22]. The focus may be less on innovation and more on enhancing revenue and sales of existing products. This aligns with evolving “product hopping” strategies in which investments focus on new indications or reformulations of existing drugs. For example, AbbVie recently expanded indications for risankizumab beyond dermatology to compete with adalimumab biosimilars [23].

Changes in research funding may also be a side effect of the increasing role of PE in the field of dermatology. Over the past decade, PE firms have rapidly acquired and consolidated medical practices, including dermatology. In 2018, PE controlled 10% of dermatology practices, representing a 349% increase from 2011 [24]. PE investment in dermatology is driven by its high payment rates, the scale of the patient population, and the field’s fragmented nature [25,26]. The decline in research payments observed in our study may reflect a broader shift in funding dynamics, with increasing reliance on PE and venture capital-backed trials reducing the pharmaceutical industry’s direct financial role [27]. This shift may reduce transparency, making it more difficult to identify potential conflicts of interest that could influence research outcomes. Further research is needed to understand whether PE’s growing presence influences the number and nature of research payments between industry and physicians within the OPP.

While the OPP has improved the transparency of industry-physician financial relationships, research payments, particularly those involving NCEs, remain poorly characterized due to limited reporting requirements [14]. Many of the highest-funded NCEs are private organizations that receive substantial industry research payments yet are not required to disclose how the funds are allocated toward equipment, research infrastructure, physician compensation, or clinical trial operations. Moreover, approximately half of all direct and associated research payments were not attributed to a covered product, obscuring whether these financial relationships influence study design, trial outcomes, clinical adoption, or purchasing decisions, as has been demonstrated for general payments [28-30]. Limited disclosure requirements for NCEs and the absence of some product attribution prevent stakeholders from fully assessing conflicts of interest, research bias, and the broader implications of industry financial relationships.

To address these gaps, comprehensive reforms to OPP reporting requirements for research payments to NCEs are needed. Future policy changes could mandate that NCEs provide itemized disclosures that 1) categorize expenditures in detail, including products, equipment, and research expenses; 2) clearly document whether funds are transferred wholly or partially to individual physicians or other entities; and 3) broaden the definition of covered products to reduce the share of research payments without an attributed product. In addition, publicly linking NCE-reported expenditures to clinical trial registry data could allow for independent verification of whether payments align with trial activity and outcomes. Together, these reforms would strengthen transparency and enable policymakers, regulators, clinicians, and patients to make more informed assessments of potential conflicts of interest and research bias in industry-sponsored studies.

Study limitations

Due to incomplete reporting by NCEs, our methods attributed total payment to the primary PI when multiple PIs were listed, which may have led to an overestimation of payments to certain dermatologist PIs. In addition, the OPP database lacks granularity in expenditure breakdowns, limiting insight into how research funds were allocated. Potential confounders such as geography or physician-specific variables beyond gender were not available. Payments from mergers or acquisitions were attributed to parent companies, possibly inflating aggregated values. 

## Conclusions

Dermatology, as a case example of a specialty with a high volume of complex financial transactions, reveals important areas for improving the transparency of industry-physician relationships. In dermatology, the majority of research payments are directed to NCEs, which have fewer reporting requirements, leaving large gaps in publicly available data in the OPP. Without clear information on whether funds are used for research expenses, physician compensation, or clinical trial operations, there is limited ability to interpret the true scope and impact of industry financial relationships. Requiring detailed reporting of NCE payment allocation for products and equipment, research expenses, and physician compensation, as well as linking reported expenditures to clinical trial registries, would help ensure that industry-physician relationships do not compromise research integrity, patient care, or public trust in healthcare systems.
